# Accessing Citrus and Soybean Flavonoids as Potential Efflux Pump Inhibitors in Drug-Resistant *Escherichia coli*

**DOI:** 10.3390/antibiotics14121229

**Published:** 2025-12-06

**Authors:** Wen-Jung Lu, Yi-Chi Huang, Ching-Yi Tai, Hong-Ting Victor Lin

**Affiliations:** 1 Department of Food Science, National Taiwan Ocean University, No. 2, Pei-Ning Road, Keelung 202, Taiwan; 2 School of Life Sciences, University of Nottingham, Queen’s Medical Centre, Nottingham NG7 2UH, UK; 3 Center of Excellence for the Oceans, National Taiwan Ocean University, No. 2, Pei-Ning Road, Keelung 202, Taiwan

**Keywords:** efflux pump inhibitors, multidrug resistance, drug transporters, flavonoids, molecular docking

## Abstract

Background/Objectives: Drug efflux pumps represent a significant challenge that contributes to the development of antibiotic resistance in bacteria. This research aimed to evaluate the flavonoids apigenin, chrysin, glycitein, and hesperetin for their potential to inhibit efflux pumps in drug-resistant *Escherichia coli*. Method: The antibacterial activity of the flavonoids was assessed using minimum inhibitory concentration (MIC) and modulation assays. Dye accumulation and efflux assays were performed to evaluate effects on efflux pump function, while membrane permeability and biofilm formation assays were also conducted. Molecular docking was used to examine interactions between the flavonoids and the AcrB efflux transporter. Results: Although the flavonoids showed limited intrinsic antibacterial activity, they enhanced the effectiveness of erythromycin, ciprofloxacin, and clarithromycin against drug-resistant *E. coli*. Apigenin and hesperetin significantly increased dye accumulation and reduced dye efflux, indicating interference with substrate translocation through efflux pumps. All compounds exhibited no effect on inner membrane permeability, while apigenin, chrysin, and glycitein inhibited biofilm formation. Docking results showed that apigenin and chrysin bind favorably within the distal binding pocket of AcrB, forming hydrophobic and π–π interactions with key aromatic residues such as Phe610 and Phe628, with binding affinities of –8.8 to –8.9 kcal/mol. Conclusions: The results suggest that apigenin and chrysin have promising efflux-pump inhibitory potential in drug-resistant *E. coli*, supporting their possible role as adjuvants to improve antibiotic efficacy.

## 1. Introduction

The rising prevalence of drug-resistant bacterial infections has increased treatment uncertainty, healthcare costs, and mortality worldwide. World Health Organization (WHO) has pointed out that drug resistance is a serious threat to human health, undermining the effectiveness of therapies for an expanding array of infections [1]. It is estimated that 4.71 million deaths were related to bacterial antimicrobial resistance, of which 1.14 million deaths were specifically attributed to antimicrobial resistance (AMR) in bacteria [2]. At the same time, antibiotic development has slowed due to scientific, financial, and regulatory challenges [3].

One of the primary mechanisms contributing the antibiotic resistance is the presence of bacterial drug efflux pumps. These active transport systems expel toxic compounds and reduce intracellular drug accumulation [4]. A notable case of this is the tripartite efflux pump system, commonly found in various Gram-negative bacteria, including AcrAB-TolC in *Escherichia coli* [5] and MexAB-OprM in *Pseudomonas aeruginosa* [6]. This system is capable of transporting a broad spectrum of antibiotics, including macrolides, fluoroquinolones, tetracyclines and is recognized as a key mechanism underlying multidrug resistance in these bacteria species.

Efflux pump inhibitors (EPIs) have therefore attracted interest as adjunctive agents capable of restoring antibiotic activity by interfere with pump function [7]. Their use in adjunctive therapy may enhance or restore the functionality of antibiotics by inhibiting efflux pumps, disrupting the assembly of these pumps or their energy supply. Several established and effective EPIs were restricted in clinical application as a result of their cytotoxic properties [8]. Due to the ongoing challenges faced in the development of EPIs, there has been a growing interest in EPIs derived from natural sources. These natural EPIs are considered promising candidates due to their diverse phytochemical profiles and low toxicity levels [9]. A wide variety of compounds with complex chemical structures are found in plants and their extracts [10]. In recent years, several studies have focused on investigating different classes of plant secondary metabolites as potential inhibitors of efflux pumps [11].

Flavonoids are prevalent secondary metabolites found in plants, particularly abundant in diets rich in fruits and vegetables, as well as in various herbal remedies. Current research suggests that flavonoids are crucial in exhibiting antibacterial properties through mechanisms such as disrupting the cytoplasmic membrane [12], interfering with energy metabolism, and suppressing nucleic acid synthesis across different microbial species. Several studies have been undertaken to assess the antimicrobial effects of flavonoids, along with an overview of the possible connections between their chemical structures and antimicrobial properties. However, most studies have focused on the efficacy of flavonoids in inhibiting the NorA efflux pump in Gram-positive bacteria, with limited information available regarding their impact on Gram-negative bacteria. In this study, we sought to explore the inhibitory potential of flavonoids on the efflux pump AcrB (also known as acridine resistance protein B) and to further enhance their application as an adjuvant in addressing against antibiotic resistance.

## 2. Results and Discussion

### 2.1. Modulation Assay for Flavonoids Against Drug-Resistant E. coli

Our MIC assays indicated that apigenin, chrysin, glycitein, and hesperetin did not exhibit notable bactericidal activity against *E. coli* Kam3-AcrB at their respective maximum soluble concentrations d As shown in Table 1, the modulation assays involved ciprofloxacin, clarithromycin, erythromycin, and tetracycline, all established as transport substrates of the AcrB efflux pump. The minimum inhibitory concentrations (MICs) for each antibiotic and the modulation factors (MFs) in the presence of the flavonoids against *E. coli* Kam3-AcrB are presented.

The MIC measurements for ciprofloxacin, clarithromycin, erythromycin, and tetracycline were 0.06, 247.5, 384, and 1.2 µg/mL, respectively. Clarithromycin MICs were reduced 2–4-fold in the presence of each flavonoid, while chrysin and glycitein decreased the MICs of erythromycin and ciprofloxacin by 2-fold. The reference efflux inhibitor PAβN demonstrated the expected modulation activity and was included as a positive control. Our modulation results demonstrated that apigenin, chrysin, glycitein and hesperetin have the potential to influence the efficacy of erythromycin, ciprofloxacin, and clarithromycin against Kam3-AcrB.

The compound apigenin exhibited synergistic activity with ampicillin and ceftriaxone against methicillin-resistant *Staphylococcus aureus* (MRSA) strains [13]. In addition, it has been revealed that several apigenin derivatives [14,15] can inhibit the activity of DNA gyrase in *E. coli* and exhibit inhibitory effects on the formation of *E. coli* biofilm, indicating its potential multifunctional capabilities against different bacterial strains. Baicalein, a flavone with weak antimicrobial properties derived from *Thymus vulgaris*, enhanced the susceptibility of the MRSA strain SA-1199B to multiple β-lactam antibiotics and ciprofloxacin, likely through inhibition of the NorA efflux pump [16]. It has also been reported to inhibit tetK-mediated efflux, thereby restoring *E. coli* sensitivity to tetracycline [17].

### 2.2. Pump Efflux Efficiency Reduced by Flavonoids

**Figure 1:**
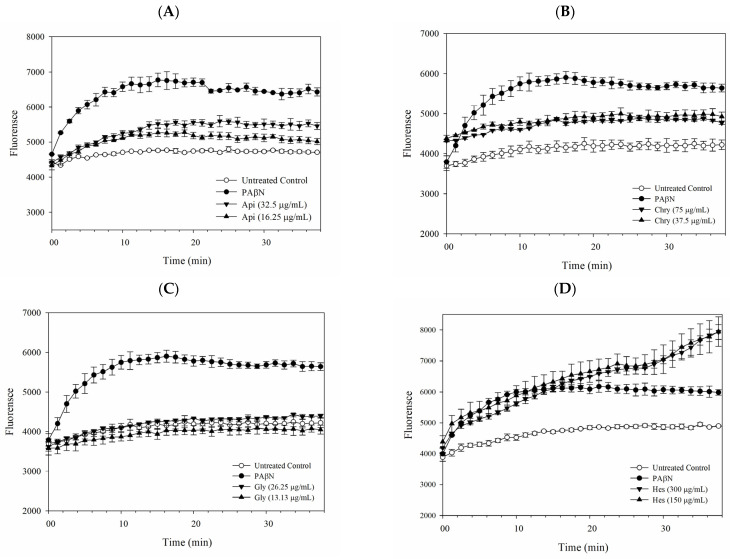
EtBr accumulation of apigenin (**A**), chrysin (**B**), glycitein (**C**), hesperetin (**D**) in Kam3-AcrB. Data are presented as mean ± SD (*n* = 3).

Fluorescent assays have been broadly applied to deepen our understanding of the efflux mechanisms of substrates in cells that express efflux pumps. The assays for accumulation and efflux were conducted to assess the inhibitory effectiveness of the efflux pump by monitoring changes in fluorescence. In this research, the accumulation and efflux processes utilize ethidium bromide (EB), recognized as one of the substrates for AcrB, along with a RND pump modulator PAβN serving as the positive control group. The accumulation resultes showed that *E. coli* Kam3-AcrB accumulated higher levels of ethidium bromide when exposed to apigenin, chrysin, and hesperetin. In comparison, glycitein displayed a fluorescence intensity similar to that of the control group, which lacked any EPIs. This observation offers indirect evidence that apigenin, chrysin, and hesperetin may interfere with the efflux mechanism of EB by Kam3-AcrB (Figure 1).

**Figure 2:**
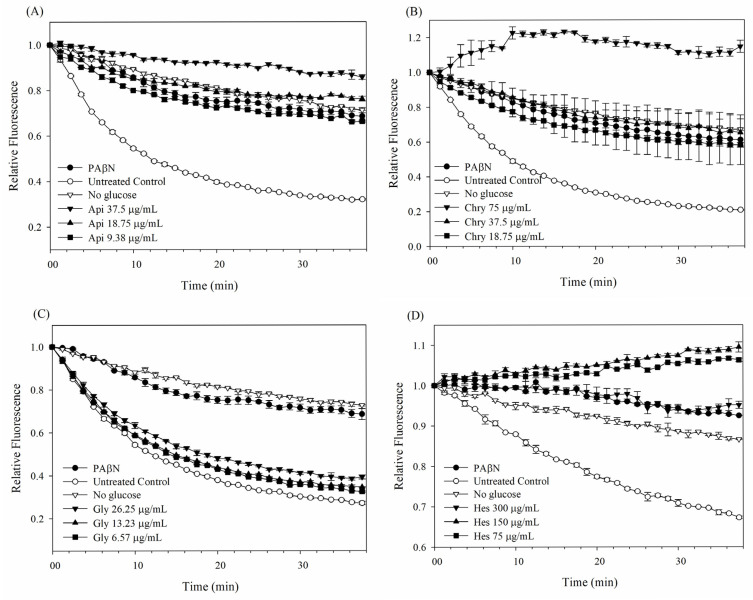
EtBr efflux inhibition assay of flavonoids in *E. coli* Kam3-AcrB. (**A**) apigenin, (**B**) chrysin, (**C**) glycitein, (**D**) hesperetin. Data are presented as mean ± SD (*n* = 3).

The possible impact of a pump efflux inhibitor on *E. coli* Kam3-AcrB was further examined by observing the efflux of EB in the presence of flavonoids. Figure 2 demonstrates a progressive reduction in EB fluorescence attributed to Kam3-AcrB control group, which reflects a continuous efflux of EB from the *E. coli* cells. In the absence of supplementary glucose for energization, the Kam3-AcrB demonstrated a greater EB fluorescence at 38 min when compared to the control group. Additionally, the presence of the pump inhibitor PAβN caused a reduction in EB efflux, suggesting it may disrupt the function of the efflux pump. The incorporation of apigenin, chrysin, and hesperetin at concentrations of MIC, 1/2 MIC, and 1/4 MIC is capable of lowering the rate at which EB fluorescence decreases in a dose-dependent manner, suggesting their potential to disrupt the efflux of EB from the cells. In contrast, glycitein did not show any EB efflux, which is consistent with our accumulation data. The findings from the dye efflux assays aligned with the results obtained from the dye accumulation assays, demonstrating that apigenin, chrysin, and hesperetin may disrupt the efflux of EB, thereby indicating its potential as an efflux pump inhibitor.

With respect to phenolic compounds, several previous investigations have revealed their interaction with efflux pumps [18,19]. The results from Brown et al. [20] suggest that the flavonoid compound apigenin exhibits a dose-dependent inhibitory effect on the dye efflux assay targeting the NorA efflux pump in *S. aureus*. In addition, flavonoid quercetin has been found to significantly inhibit the AcrB efflux activity along with the *S. aureus* strains that contain the TetK pump and NorA efflux pump [21,22]. Irianti et al. [23] isolated prenylated isoflavonoids from *Fabaceae* plants and showed that these compounds significantly inhibit the NorA efflux pump in *S. aureus*, as evidenced by enhanced ethidium bromide accumulation and reduced efflux. Waditzer and Bucar [19] reviewed that numerous flavonoid subclasses inhibit bacterial efflux pumps such as NorA, MepA, and AcrAB-TolC, typically leading to decreased MICs of co-administered antibiotics and increased intracellular accumulation of ethidium bromide or fluoroquinolones.

### 2.3. Inner Membrane Permeability and Biofilm Formation

**Figure 3:**
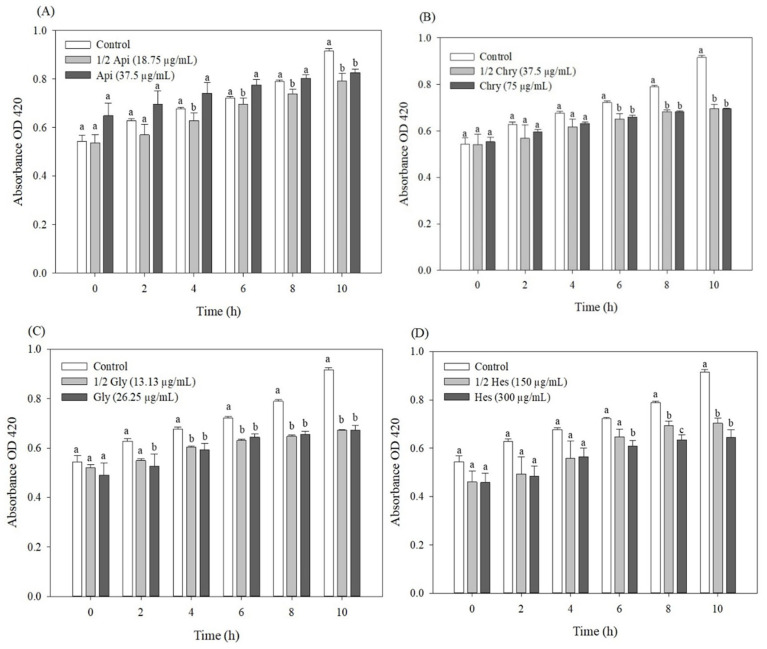
Effect of apigenin (**A**), chrysin (**B**), glycitein (**C**), and hesperetin (**D**) on the inner membrane permeability of Kam3-AcrB. Data are presented as mean ± SD (*n* = 3). Different letters above the bars within the same group indicate significant differences (*p* < 0.05).

It is well established that reduced membrane permeability and biofilm formation contribute to antibiotic resistance by limiting antibiotic entry or creating protective barriers that diminish drug efficacy [24,25]. Flavonoids have been reported to interfere with these processes by reducing bacterial adhesion, suppressing biofilm formation, altering porin expression, and interacting with the phospholipid bilayer to affect membrane integrity [26]. To further explore the mechanisms underlying the inhibition caused by flavonoids, the ONPG assay and biofilm inhibition were used to evaluate how apigenin, chrysin, glycitein, and hesperetin affect the permeability of the inner membrane and the inhibition of biofilm in Kam3-AcrB. Ortho-nitrophenyl-β-galactoside (ONPG) is a chromogenic substrate that can diffuse across the outer membrane through non-specific porins, entering the cytoplasm solely when the inner membrane becomes permeabilized. The production of ONP was observed by determining the increase in absorbance over time, as shown in Figure 3. Permeability was evaluated by utilizing an equivalent 3.5% DMSO concentration in the control group to match the solvent used to dissolve the flavonoid. A notable rise in the permeability of the inner membrane was observed following a 10 h incubation, both with and without the application of flavonoid treatment. The flavonoid tested at both 1/2 MIC and MIC displayed a similar level of inner membrane permeability; in addition, it did not increase the inner membrane permeabilization in comparison to Kam3-AcrB that was untreated with any flavonoids, indicating the flavonoids did not alter the inner membrane permeability.

A previous investigation revealed that the application of apigenin alone did not lead to an increase in inner membrane permeability; but when used in conjunction with ceftazidime, it has the potential to cause damage to the cytoplasmic membrane in ceftazidime-resistant *E. cloacae* strains [27]. Further research revealed that the ethanolic extracts from *Frangula alnus* Mill are capable of reducing the membrane permeability of both probiotic and environmental bacteria, potentially through the regulation of cellular transport processes [28].

**Figure 4:**
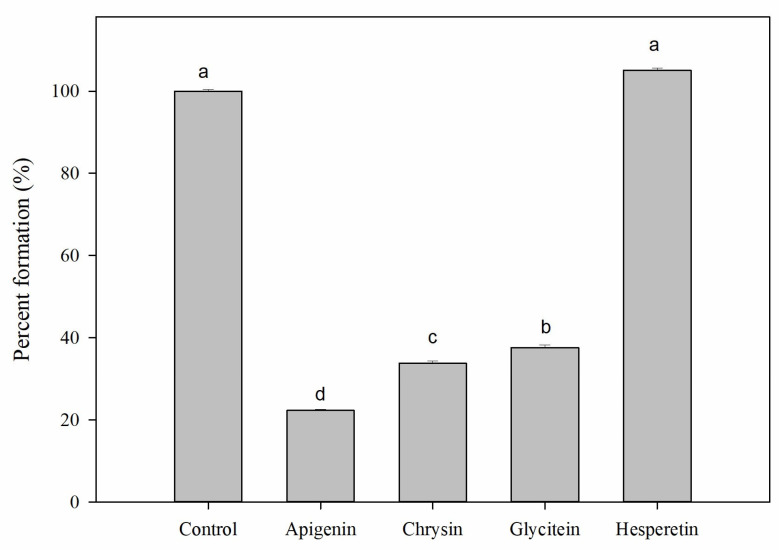
Effect of apigenin chrysin, glycitein, hesperetin on biofilm inhibition in *E. coli* Kam3-AcrB. Data are presented as mean ± SD (*n* = 3). Different letters above the bars within the same group indicate significant differences (*p* < 0.05).

Bacterial biofilm is defined as a community of living microorganisms that are engaged in the production of a self-generated extracellular matrix. Efflux pumps contribute to biofilm formation through several mechanisms, including mediating initial adherence, extruding harmful substances, and regulating genes involved in biofilm development [29]. EPIs have been reported to prevent biofilm formation in certain bacteria, offering a potential strategy for treating infections caused by pathogens [30,31]. As shown in Figure 4, the results demonstrate that the application of apigenin, chrysin, and glycitein at their 1/2 MIC has led to a reduction in biofilm formation by 77.7%, 66.24%, and 62.42%, respectively, when compared to the control group. In contrast, the compound hesperetin exhibited no inhibitory activity. It has been observed that the inhibition of efflux pump systems might lead to an increased disruption of biofilm formation. Numerous studies have indicated that natural EPIs derived from plants could potentially interfere with the bacterial quorum sensing or directly disrupt the biofilm formation.

A natural flavonoid compound Baicalin from the roots of *Scutellaria baicalensis*, has shown to reduce biofilm formation in *Staphylococcus saprophyticus* by inhibiting the MsrA efflux pump [32]. Lopes et al. [33] have demonstrated that flavonoids myricetin, hesperetin and phloretin have an inhibitory effect on biofilm formation while promoting the overexpression of the msrA and norA efflux pumps in *S. aureus*. Evidence shows that chemical inhibition of efflux pumps has been proven to diminish the transcription of matrix-producing genes, which subsequently disrupts the ability of bacteria to develop mature biofilms [34].

### 2.4. Postantibiotic Effect of Flavonoids Coupled with Clarithromycin on E. coli Kam3-AcrB

The post-antibiotic effect (PAE) refers to the continued sustained inhibition of bacterial growth that continues even after an antimicrobial agent is removed. This effect can be assessed by determining the period during which the target bacteria do not proliferate after the antibiotic treatment has ended. As shown in Table 2, the PAE values of clarithromycin alone at 1/2 × MIC and 2 × MIC for Kam3-AcrB were determined to be 0.14 ± 0.08 and 0.21 ± 0.00, respectively. The PAE values for the tested compounds—apigenin, chrysin and glycitein—exhibit no significant difference when combined with 1/2 × MIC and 2 × MIC clarithromycin. Conversely, the combination of hesperetin with 1/2 × MIC and 2 × MIC clarithromycin raises the PAE for Kam3-AcrB, resulting in a minor increase in PAE of 0.32 ± 0.03 and 0.37 ± 0.02 at 1/2 × MIC and 2 × MIC of clarithromycin. Lu et al. [35] reported that the phenolic compound ethyl 3,4-dihydroxybenzoate (EDHB) could have a minor effect on increasing the PAE of erythromycin for *E. coli* that expresses the AcrB efflux pump, from 0.3 h to 0.41 h. Glabridin, a type of flavonoid found in licorice root, has been identified to increase the PAE in *S. aureus* isolates from 0.8, 1.4 and 2.9 h to 2.42, 3.44 and 4.57 h when combined with 1/4 × MIC, 1/2 × MIC, and MIC of norfloxacin, respectively [36]. So far, the effectiveness of EPIs in prolonging the PAE of antibiotics targeting Gram-negative bacteria has also been reported in few studies.

### 2.5. In Silico Prediction of Molecular Interaction

Structural studies of the *Escherichia coli* resistance–nodulation–division (RND) transporter AcrB have revealed essential mechanistic features that underlie its multidrug efflux activity. High-resolution crystallography has identified distinct structural domains, including periplasmic entry channels, multiple drug-binding pockets, and a substrate extrusion pathway coordinated through a functional rotation mechanism [37]. In this study, molecular docking analysis performed using AutoDock Vina 1.2.0 demonstrated that the tested compounds exhibit variable affinities toward the distal and proximal binding pockets of AcrB, suggesting selective recognition and accommodation within the transporter [38]. By targeting the tight conformation of the AcrB monomer (PDB 4DX5), our investigation revealed diverse interaction profiles among key residues in both binding pockets, highlighting the structural complexity and adaptability of the AcrB drug-binding architecture.

**Figure 5:**
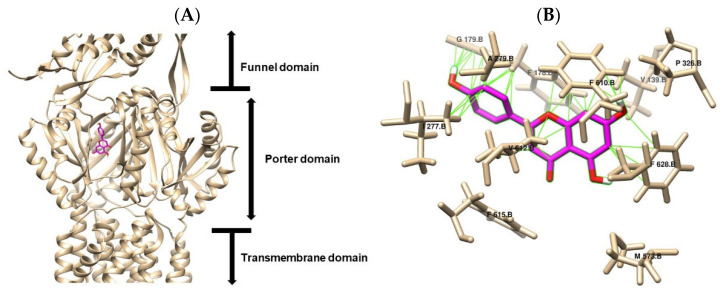
Binding interaction models of apigenin (pink) within the AcrB efflux pump. (**A**) Overall binding pocket architecture showing apigenin positioning within the AcrB efflux pump. (**B**) Detailed view of key molecular interactions between apigenin and interacting residues. The apigenin are colored in magenta.

**Figure 6:**
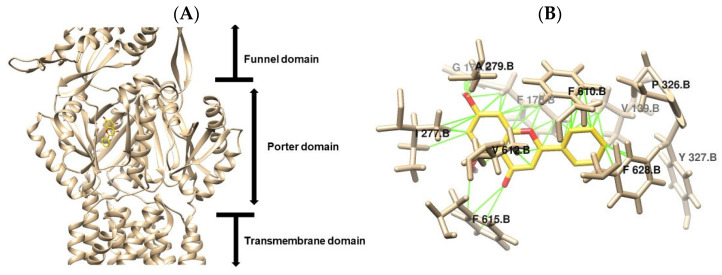
Binding interaction models of chrysin (yellow) within the AcrB efflux pump. (**A**) Overall binding pocket architecture showing chrysin positioning within the AcrB efflux pump. (**B**) Detailed view of key molecular interactions between chrysin and interacting residues. The chrysin are colored in yellow.

The results of the molecular docking performed with Autodock Vina demonstrated varying levels of binding affinity and interactions among the tested flavonoids for the drug-binding pockets of the *E. coli* RND drug transporter AcrB. Figure 5 and Figure 6 illustrate the molecular docking poses of apigenin and chrysin, respectively, within the binding cavity of the AcrB efflux pump (PDB ID: 4DX5). Both flavonoids were located within the porter domain of AcrB, specifically occupying the distal binding pocket (DBP), a region rich in hydrophobic and aromatic residues known to accommodate diverse substrates. As shown in Figure 5A, apigenin fitted within the hydrophobic cavity of the DBP. The detailed interaction map (Figure 5B) highlights key interactions with residues Val139, Phe178, Gly179, Ile277, Ala279, Phe610, Val612, Phe615, and Phe628. These contacts result primarily from hydrophobic packing and π–π interactions, with aromatic residues such as Phe610, Phe615, and Phe628 anchoring the flavonoid ring scaffold. Similarly, Figure 6A,B shows that chrysin occupies a binding region nearly identical to apigenin, consistent with their structural similarity. Chrysin also interacts with Val139, Phe178, Gly179, Ile277, Ala279, Phe610, Val612, Phe615, and Phe628, as reported in Table 3, but with the additional involvement of Tyr327, suggesting a marginally stronger stabilization via additional aromatic π–π interaction.

Docking results summarized in Table 3 show comparable binding free energies for apigenin (–8.8 kcal/mol) and chrysin (–8.9 kcal/mol), each forming 40–41 intermolecular contacts. Notably, the π–π interactions and van der Waals forces observed between apigenin and AcrB, as well as between chrysin and AcrB, align with occupancy of the distal binding pocket, where phenylalanine-rich residues play a dominant role in ligand stabilization [39]. Lu et al. [40] demonstrated through molecular docking analysis that PAβN, a well-characterized AcrB inhibitor, forms hydrogen bonds with AcrB residues Ser134 and Gln176, both situated in the PN2 subdomain of AcrB along the pathway to the binding pocket [41]. This interaction pattern was not observed at the docking interface between flavonoids and AcrB, and such polar anchors may prolong residence time and enhance pump inhibition beyond what ΔG alone predicts.

While our in silico and in vitro findings provide initial evidence of flavonoid interactions with AcrB, further validation is required. Future studies employing ex vivo infection models or co-crystallography will be essential to confirm the physical binding interactions and to assess the therapeutic relevance of these compounds in more complex biological systems.

## 3. Materials and Methods

### 3.1. Bacterial Strains, Constructs, Media and Chemicals

The *E. coli* Kam3 (DE3) strain, which lacks acrB, was used to carry out assays focused on drug susceptibility and modulation [29]. Simultaneously, the Kam3 strains harboring the pSYC plasmid with the *acrB* gene was employed for a wider array of analyses. Cultivation and microdilution experiments were performed utilizing Luria–Bertani (LB) broth and Mueller–Hinton (MH) broth. Erythromycin, ciprofloxacin, clarithromycin, tetracycline, apigenin, chrysin, glycitein and hesperetin were obtained from Sigma-Aldrich (St. Louis, MO, USA). Apigenin, chrysin, glycitein and hesperetin stock were prepared by dissolving them in 3.5% DMSO at concentrations of 62.5, 125, 43.75, and 1000 μg/mL, respectively, followed by dilution in PBS buffer and media for the experiments of this study.

### 3.2. Minimum Inhibitory Concentration (MIC) Determination

Minimum inhibitory concentration (MIC) testing was conducted following previously established methodologies incorporating a few minor alterations [42]. The MICs for apigenin, chrysin, glycitein, and hesperetin were determined through a Mueller–Hinton broth microdilution assay utilizing *E. coli* strains, both susceptible and resistant, cultivated from logarithmic-phase cells at 10^5^ CFU/mL. Each drug was diluted with MH broth in a two-fold serial dilution series in a round-bottomed 96 well plate. After a 112 h incubation at 37 °C, the MIC was assessed following CLSI guidelines. The highest concentrations tested for apigenin, chrysin, glycitein and hesperetin were 62.5, 125, 43.75 and 1000 μg/mL, respectively.

### 3.3. Modulation Assays

The potentiating efficacy of flavonoids in combination with antibiotics was evaluated through a modulation assay [43]. Briefly, antibiotics and the compounds being tested were diluted in flat-bottomed 96-well microplates, utilizing a series of twofold dilutions from the MIC across two dimensions. These were mixed with a bacterial culture of 105 CFU/mL and incubated at 37 °C for 12 h. The modulation factors for each antibiotic, along with their EPI concentrations were recorded.

### 3.4. Dye Accumulation Assay

Following earlier methods with modifications [44,45], the accumulation of ethidium bromide (EB) in *E. coli* was evaluated using mid-log-phase cultures cultivated in MH broth. The cells were harvested through centrifugation (5000× *g*, 5 min, 4 °C), subsequently washed twice in PBS with a pH of 7.4, and finally resuspended and diluted in PBS to reach an OD_600_ of 0.6. Following this, the cell suspension was placed in a 96-well plate and incubated with filter-sterilized glucose (25 mM) for 3 min at room temperature. After the addition of EB (25 μM), fluorescence was continuously recorded for a duration of 38 min. The excitation and emission wavelengths for EB were set at 520 nm and 600 nm. The individual impacts of PAβN (final concentration of 20 μg/mL), apigenin, chrysin, glycitein, and hesperetin were evaluated by incubating each with the bacterial suspension prior to fluorescence measurement.

### 3.5. Efflux Inhibition Assay

The dye efflux assay was executed based on established protocols with slight modifications [46]. Mid-log-phase cultures of *E. coli* Kam-AcrB, which were grown in MH broth, underwent centrifugation at 5000× *g* for 5 min at a temperature of 4 °C. Following the centrifugation process, the cells were washed twice with PBS and subsequently diluted in fresh PBS until the OD_600_ was set to 0.6. After an 8 h incubation at RT, the *E. coli* cells were exposed to EB at a concentration of 3 μM for 30 min. The cells were then resuspended twice in PBS buffer and transferred to 96-well plates containing filter-sterilized glucose (25 mM), PAβN (20 μg/mL), and various concentrations of apigenin, chrysin, glycitein, and hesperetin at RT, with fluorescence being monitored over a period of 38 min.

### 3.6. Postantibiotic Effect Assay

As described in prior research [47], *E. coli* Kam3-AcrB was cultured at 37 °C until it reached the mid-log growth stage. The bacterial suspension was then divided into several experimental groups: one control group, two groups treated with clarithromycin at half the minimum inhibitory concentration or double the minimum inhibitory concentration, and further groups that received the same clarithromycin doses in combination with apigenin (62.5 μg/mL), chrysin (125 μg/mL), glycitein (43.75 μg/mL), and hesperetin (500 μg/mL). After a 2 h incubation, the bacterial culture was transferred into fresh LB broth at a thousand-fold dilution, and the cell counts (CFU/mL) were quantified hourly through plate counting until the cell counts reached ten times the initial counts. The duration required for the bacterial counts to increase by 1 log in each group can then be determined (PAE = T − C).

### 3.7. Inner Membrane Integrity Assay

The evaluation of the membrane permeabilisation of *E. coli* in response to flavonoids was determined by quantifying the release of β-galactosidase. This measurement utilized the chromogenic substrate ortho-Nitrophenyl-β-galactoside (ONPG), which is hydrolyzed by the cytoplasmic enzyme β-galactosidase, producing ortho-nitrophenol (ONP, yellow) [48]. Briefly, *E. coli* Kam3-AcrB cells were grown in LB broth medium until the OD600 reached 0.6. The cell pellet was collected via centrifugation at 6000× *g* for 10 min. The cells were then resuspended in PBS and adjusted to a final OD600 of 0.5. The cell suspensions were incubated with ONPG (1.5 mM), apigenin, chrysin, glycitein, and hesperetin at 37 °C for 10 h, and the fluorescence was monitored using a microplate reader every 2 h under 420 nm. All of the compounds were dissolved in 3.5% of DMSO.

### 3.8. Biofilm Inhibition

The measurement of biofilm formation was carried out following established protocols with some modification [48]. Fresh cultures of *E. coli* Kam3-AcrB in the log phase were incubated overnight at 37 °C in 96-well flat-bottom microplates with apigenin, chrysin, glycitein, and hesperetin. The monitoring of biofilm formation was performed using crystal violet staining. After the media was removed, the biofilm was rinsed three times with sterile water and subsequently fixed with ethanol. Subsequently, the biofilms were stained with 200 μL of a 0.1% crystal violet solution for 30 min, washed three times with milliQ water, and the biofilm biomass was quantified by resolubilizing the crystal violet in 200 μL of 75% ethanol for 15 min, with absorbance measured at 595 nm.

### 3.9. Molecular Docking

Autodock Vina [38] was applied for docking studies utilizing its default settings, while UCSF Chimera version 1.16 [49], was employed for visualization purposes. The 1.9 Å crystal structure of AcrB (PDB: 4DX5) was obtained from the Protein Data Bank to serve as the docking target [50]. The structures of the ligands—apigenin, chrysin, glycitein, and hesperetin—were sourced from PubChem (https://pubchem.ncbi.nlm.nih.gov) and later transformed into PDB files through the use of Chimera 1.16. It is recognized that AcrB can exist in three distinct conformational states during the substrate transport mechanism; thus, the docking region was selected within the distal binding pocket (DBP) and proximal binding pocket (PBP) of the tight state of the AcrB monomer with a search space dimension of 30 Å × 30 Å × 30 Å.

### 3.10. Statistical Analysis

Data analysis was performed using SPSS version 12 (Chicago, IL, USA), with results presented as the mean ± standard deviation. To assess significant variation among treatments, a one-way ANOVA was applied at a significance level of *p* < 0.05. Multiple mean comparisons were subsequently analyzed using Tukey’s test.

## 4. Conclusions

In this study, we assessed the flavonoids for their EPI activity against drug-resistant *E. coli*. Our findings suggest that apigenin and chrysin could potentiate the antibiotic activity against drug-resistant *E. coli* by interfering with the efflux pump AcrB. Moreover, they did not appear to alter the permeability of the bacterial inner membrane and also inhibited biofilm formation. Despite the fact that hesperetin demonstrated its capability as an EPI against drug-resistant *E. coli*, the effective doses utilized in this study were considered as a high concentration, which poses a risk for future applications due to potential toxicity. To conclude, it is essential to conduct further in vivo experiments and pharmacokinetic studies for apigenin and chrysin in the future to validate clinical efficacy.

## Figures and Tables

**Table 1:** MIC modulation assay of apigenin, chrysin, glycitein and hesperetin with various antibiotics for *E. coli* Kam3-AcrB.

Compound	Antibiotics	MIC (μg/mL)		MF
		Alone	Combination (Antibiotic/Flavonoid) *	
Apigenin	Ciprofloxacin	0.06	0.06/62.5	1
	Clarithromycin	247.5	61.87/62.5	4
	Erythromycin	384	384/62.5	1
	Tetracycline	1.2	1.2/62.5	1
Chrysin	Ciprofloxacin	0.06	0.06/125	1
	Clarithromycin	247.5	123.75/125	2
	Erythromycin	384	192/125	2
	Tetracycline	1.2	1.2/125	1
Glycitein	Ciprofloxacin	0.06	0.03/21.87	2
	Clarithromycin	247.5	123.75/43.75	2
	Erythromycin	384	384/43.75	1
	Tetracycline	1.2	1.2/43.75	1
Hesperetin	Ciprofloxacin	0.06	0.06/250	1
	Clarithromycin	247.5	123.75/250	2
	Erythromycin	384	384/250	1
	Tetracycline	1.2	1.2/250	1
PAβN	Ciprofloxacin	0.06	0.06/20	1
	Clarithromycin	247.5	30.94/10	8
	Erythromycin	384	48/20	8
	Tetracycline	1.2	1.2/20	1

MF, modulation factor. * Values in the combination column denote (MIC of antibiotic)/(concentration of flavonoid).

**Table 2:** Post-antibiotic effect of flavonoids in combination with clarithromycin on *E. coli* Kam3-AcrB.

Regimen	Mean PAE (h) ± SD	
	1/2 MIC	2 MIC
Clarithromycin	0.14 ± 0.08 ^b^	0.21 ± 0.00 ^b^
Clarithromycin + Apigenin	0.1 ± 0.09 ^b^	0.14 ± 0.09 ^b^
Clarithromycin + Chrysin	0.2 ± 0.09 ^b^	0.21 ± 0.05 ^b^
Clarithromycin + Glycitein	0.06 ± 0.03 ^b^	0.09 ± 0.01 ^b^
Clarithromycin + Hesperetin	0.32 ± 0.03 ^a^	0.37 ± 0.02 ^a^

PAE, post-antibiotic effect. 1/2 MIC, 123.75 µg/mL. 2 MIC, 495 µg/mL. Apigenin, 62.5 µg/mL. Chrysin, 125 µg/mL. Glycitein, 43.75 µg/mL. Hesperetin, 500 µg/mL. Data are expressed as mean ± SD (*n* = 3). Different letters in the same column indicate significant differences (*p* < 0.05).

**Table 3:** Molecular docking of the flavonoids against transporter AcrB (PDB 4DX5).

Dock Parameters	Flavonoids	
	Apigenin	Chrysin
Interaction Residues	V139 ^a^, F178 ^ab^, G179 ^a^, I277 ^a^, A279 ^a^, P326, M573, F610 ^ab^, V612 ^a^, F615 ^ab^, F628 ^ab^	V139 ^a^, F178 ^ab^, G179 ^a^, I277 ^a^, A279 ^a^, P326, Y327 ^b^, F610 ^ab^, V612 ^a^, F615 ^ab^, F628 ^ab^
Hydrogen bonds	-	-
Inter-molecules contacts	40	41
Binding free energy (kcal/mol)	−8.8	−8.9

^a^ van der Waals contact distance: 2.8–4 Å. ^b^ π–π Interactions: 4.5–7.0 Å.

## Data Availability

The raw data supporting the conclusions of this article will be made available by the authors on request.
